# Editorial: Women in aging and the immune system

**DOI:** 10.3389/fragi.2024.1386626

**Published:** 2024-03-05

**Authors:** Jenna M. Bartley, Anshu Agrawal

**Affiliations:** ^1^ School of Medicine, University of Connecticut, Farmington, CT, United States; ^2^ Department of Medicine, Division of Basic and Clinical Immunology, University of California, Irvine, Irvine, CA, United States

**Keywords:** immune dysfunction, senolytic, metformin, inflammaging, aging

Women scientists have continued to excel in science, despite facing various challenges and barriers. In recent years, organizations and initiatives promoting gender equality and diversity in STEM fields have emerged to support and empower women in science. Despite progress, gender disparities still exist in certain scientific fields, highlighting the ongoing need for efforts to promote inclusivity and equality in science. This issue focuses on Women in aging and immunity to celebrate the successes of women in the field and to serve as an inspiration to both women and men.

The manuscript by Mukherjee et al. identifies γδ T cells found in the visceral adipose tissues (VAT) as significant contributor to inflammaging. Using elegantly designed studies that utilize T cell receptor delta knockout (TCRδ KO) mice and isochronic parabiotic pairs of mice, they show that γδ T are tissue resident cells and their number increases from middle age onwards age in the VAT because of a reduction in apoptosis. These long-lived γδ T cells not only contribute to systemic and local inflammation but also affect the metabolic phenotype of cells. These insights into the changes in fat tissue and its contribution to age-related inflammaging are essential for design of novel therapies. In addition to inflammation, immune dysfunction is also a hallmark of aging and Kell et al. provide a comprehensive review of how DNA damage contributes to dysregulated immune function with aging. While declines in immune function with age are well-described, the mechanisms that underly this dysfunction are less understood. DNA damage markers increase in immune cells with age, and more recently, persistent DNA damage has been shown to have a causal link to overall organismal aging and age-related immune dysregulation. Kell et al. review the current literature on how DNA damage contributes to cellular senescence in both hematopoietic and non-hematopoietic cells, and more specifically examine the current literature on increased DNA damage within immune cells, focusing on T cells. Finally, the review provides insight into potential “geroprotective” strategies to target age-related DNA damage and improve immune responses with aging. Overall, this review provides an excellent up-to-date synopsis of the current understanding of how DNA damage impacts immune function with aging.

The manuscripts by Martin et al., Torrance et al., Stancu et al., and Tonutti et al. focus on improving age-related inflammation, immune dysfunction and associated diseases. Martin et al. provide a comprehensive review on the action of Metformin, an anti-diabetic drug that has emerged as an anti-aging molecule. The review underscores the advantageous effects of Metformin in bolstering immune responses against both SARs-CoV-2 and influenza infections. Retrospective review of data suggests that employing Metformin is linked with a decreased likelihood of experiencing severe COVID-19. Furthermore, Metformin appears to augment immune responses and the formation of immune memory in response to COVID-19 and influenza vaccines, suggesting its potential in enhancing immunological resilience. The potential of two other existing drugs, dasatinib and quercetin, that together function as senolytics is investigated on improving anti-influenza responses by Torrance et al. The study notes that despite previous findings indicating that senolytic drugs improve CD4 differentiation, they did not observe a positive impact of these senolytics on immune responses against influenza-including antibody and memory responses. This raises a crucial point regarding senolytics and suggests that the reduction of senescent cells may not always yield beneficial effects on immune function. Additionally, the study suggests that different combinations of senolytics may need to be tested to investigate their effects on immune responses, alongside their action as senolytics. This underscores the complexity of the relationship between senescent cells and immune function, highlighting the need for further research in this area. Stancu et al. take a different approach to improve vaccine responses in older adults. They talk about implementing a life-course approach to immunization. Most WHO and UNESCO programs are geared towards improving vaccination in pediatric populations. They suggest redefining of the policies to also focus on older adults (≥60 years old) in the immunization programs. This is important as the vaccination rates are sub optimal in older adults in several developing countries. The review by Tonutti et al. focus on another important disease of aging, osteoarthritis (OA). OA is a devasting disease that significantly affects the mobility of older adults. There are no effective treatments except maybe knee or joint replacement surgery which also is not a permanent solution. This review highlights the potential benefits of using platelet rich plasma (PRP) that is enriched in molecules contained in platelet granules including cytokine, growth factor, etc. They discuss that possible inhibition of IL-1β and Wnt signaling by PRP may underly its beneficial effects. The use of PRP to inhibit inflammation and improve metabolic pathways in other diseases need to be explored.

Calabro et al. discussion highlights the growing recognition of sex differences in immune responses, where females often exhibit enhanced responses to viral and other infections, while males tend to have a reduced risk for autoimmune diseases. Importantly, the authors extend this observation to the aging process, emphasizing that these sex-based differences persist into older age. They also delve into the influence of socio-cultural norms associated with gender, suggesting that these norms may contribute to or exacerbate the observed differences in immune function between males and females as they age. This underscores the importance of considering both biological and sociocultural factors when studying immune responses and aging in different genders.

In summary, the research showcased here highlights the rich diversity of investigations being performed by women scientists spanning Aging and the Immune System. The manuscripts elucidate advancements in our understanding of immune dysfunction and inflammatory mechanisms associated with aging. Furthermore, they illuminate potential therapeutic strategies aimed at ameliorating these age-related challenges.

